# Propanil Exposure Induces Delayed but Sustained Abrogation of Cell-Mediated Immunity through Direct Interference with Cytotoxic T-Lymphocyte Effectors

**DOI:** 10.1289/ehp.8774

**Published:** 2006-03-13

**Authors:** James M. Sheil, Marc A. Frankenberry, Todd D. Schell, Kathleen M. Brundage, John B. Barnett

**Affiliations:** Department of Microbiology, Immunology, and Cell Biology, West Virginia University School of Medicine, Morgantown, West Virginia, USA

**Keywords:** alloreactive CTLs, antigen presentation, cell-mediated immunity, cytotoxic T lymphocyte, propanil

## Abstract

The postemergent herbicide propanil (PRN; also known as 3,4-dichloropropionanilide) is used on rice and wheat crops and has well-known immunotoxic effects on various compartments of the immune system, including T-helper lymphocytes, B lymphocytes, and macrophages. It is unclear, however, whether PRN also adversely affects cytotoxic T lymphocytes (CTLs), the primary (1°) effectors of cell-mediated immunity. In this study we examined both the direct and indirect effects of PRN exposure on CTL activation and effector cell function to gauge its likely impact on cell-mediated immunity. Initial experiments addressed whether PRN alters the class I major histocompatibility complex (MHC) pathway for antigen processing and presentation by antigen-presenting cells (APCs), thereby indirectly affecting effector function. These experiments demonstrated that PRN does not impair the activation of CTLs by PRN-treated APCs. Subsequent experiments addressed whether PRN treatment of CTLs directly inhibits their activation and revealed that 1° alloreactive CTLs exposed to PRN are unimpaired in their proliferative response and only marginally inhibited in their lytic activity. Surprisingly, secondary stimulation of these alloreactive CTL effectors, however, even in the absence of further PRN exposure, resulted in complete abrogation of CTL lytic function and a delayed but significant long-term effect on CTL responsiveness. These findings may have important implications for the diagnosis and clinical management of anomalies of cell-mediated immunity resulting from environmental exposure to various herbicides and other pesticides.

Numerous studies concerning the health-related effects of environmental toxicants demonstrate that the immune system, in addition to other organ systems including the reproductive, nervous, pulmonary, and circulatory systems, is often compromised (Carpy et al. 2000; Costa 1997). Our understanding of such adverse immunologic effects, however, is largely limited to the immediate and early consequences after exposure to such agents. The principal contribution of this present article to the field of immunotoxicology research is its demonstration that the potential long-term impact of propanil (PRN) exposure on cell-mediated immunity is far more severe than its short-term consequences. This delayed appearance of irreversible PRN-induced immunotoxic effects may be important for diagnostic and therapeutic measures in assessing exposure to environmental toxicants in general.

PRN is a postemergent herbicide used extensively around the world in the cultivation of rice and wheat crops. Its particular effectiveness is due to the high level of acylamidase expression in a rice plant that allows it to detoxify PRN, whereas common grass-type weeds lack this enzyme and are killed by this herbicide (Matsunaka 1968). PRN is routinely applied several times during a growing season without detrimental effects to the plant (Casida and Lykken 1969; Smith 1961), with 3–6 lb/acre applied annually in the United States (Costa 1997; Matsunaka 1968). Thus, a high environmental exposure of humans to PRN normally occurs as an occupational risk.

Earlier reports by Barnett and co-workers (Barnett et al. 1992; Barnett and Gandy 1989; Frost et al. 2001; Theus et al. 1993; Xie et al. 1997; Zhao et al. 1995, 1998) indicated that PRN exposure results in adverse effects on most compartments of the immune system, including macrophages, B lymphocytes, and T-helper lymphocytes. Curiously, however, there appeared to be little, if any, effect on cellular immunity mediated by cytotoxic T-lymphocyte (CTL) effectors (Barnett et al. 1992; Barnett and Gandy 1989).

Given that the responsiveness of the other immune compartments examined is inhibited by PRN exposure, we hypothesized that acute PRN exposure might yet impair CTL function, albeit in a manner that is initially difficult to detect under the *in vitro* conditions used. To test this hypothesis we considered that the adverse immunotoxic effects of PRN exposure on cell-mediated immunity might be observed in one or more of three parameters: *a*) presentation of peptide antigen to CTLs by antigen-presenting cells (APCs), *b*) proliferation and differentiation of CTLs, and/or *c*) functional lytic response of activated CTL effectors.

The immune activation and functional responsiveness of CTLs can be examined and assessed independently *in vitro*. CTL activation is based on the capacity of APCs to efficiently process and present peptide antigens to CTLs and thus indirectly affects CTL responsiveness. Conversely, the functional lytic response of CTLs emerges as a result of the differentiation of naive CD8^+^ T cells into effector CTLs capable of responding through lysis of the target cell, thereby serving as a direct measure of CTL activation.

In this present article, we demonstrate three important consequences of PRN exposure on the *in vitro* parameters of CTL activation and their functional activity as effectors of cell-mediated immunity: *a*) antigen presentation to CTLs is not impaired, *b*) the functional lytic activity of primary (1°) CTLs is only marginally impaired, and *c*) upon restimulation of 1° CTLs in the absence of PRN, the secondary (2°) CTL response is completely abrogated. On the basis of these observations, we conclude that the immunotoxic effects of PRN exposure on CTLs are delayed in their appearance and directly impair the functional activity of these effectors of cell-mediated immunity. These results may have serious and important direct implications for both diagnosis and clinical management of the acute and chronic effects of PRN exposure. Furthermore, these findings warrant examining similar acute versus delayed exposure effects with respect to the immunotoxic potential of other environmental toxicants.

## Materials and Methods

### Animals

In this study we used C57BL/6 (B6; H-2^b^) and BALB/c (H-2^d^) female mice 10–12 weeks of age from Charles River Breeding Laboratories, Inc. (Wilmington, MA) or from our own breeding colony at the West Virginia University Health Sciences Vivarium. All animals used in this study were treated humanely and with regard for alleviation of suffering.

### Cell lines

Two tumor cell lines, designated P815 (H-2^d^) and EL4 (H-2^b^), were used as targets for alloreactive cytotoxic T cells. The EL4 cell line expresses class I H-2^b^ molecules and is derived from a B6 lymphoma originally induced in a C57BL/6N mouse by 9,10-dimethyl-1,2-benzanthracene (Gorer 1950). P815 is a cell line derived from a mastocytoma in DBA/2 (H-2^d^) mice, and it expresses class I H-2^d^ molecules (Plaut et al. 1973; Ralph and Nakoinz 1974). Both the EL4 and P815 cell lines have been used extensively by us and others as suitable targets for lysis in cytotoxic T-cell assays. N1 is derived from EL4 cells transfected with the vesicular stomatitis virus nucleoprotein (VSB-N) gene (Puddington et al. 1986).

### Monoclonal antibodies and fluorescence activated cell sorting analysis

We used the following H-2K^b^-specific monoclonal antibodies (mAb): 5F1 (Sherman and Randolph 1981), Y-3 (Jones and Janeway 1981), EH144 (Bluestone et al. 1985; Geier et al. 1986), Y-25 (Jones and Janeway 1981), and 28-13-3 (Ozato and Sachs 1981). Fluorescein isothiocyanate goat anti-mouse immunoglobulin (heavy- and light-chain–specific) was purchased from Southern Biotechnology Associates, Inc. (Birmingham, AL).

### Citric acid treatment of APCs

The acid treatment protocol used in these studies to strip the APC cell surfaces of class I peptide/major histocompatibility complex (pMHC) complexes is essentially the same as that described by Sugawara et al. (1987), as modified by Storkus et al. (1993). Briefly, APCs are *a*) collected and pelleted by centrifugation; *b*) resuspended in 0.5 mL citrate-phosphate buffer, pH 3.0 (citrate-phosphate buffer consists of a 1:1 mixture of 0.263 M citric acid, pH 1.8, and 0.123 M Na_2_HPO_4_); *c*) incubated in citrate-phosphate buffer for 1 min at room temperature; *d*) resuspended in 10 mL RP-10 media, pelleted, and washed with Hank’s balanced salt solution; and *e*) resuspended to appropriate concentration in RP-10 media. RP-10 tissue culture media consists of RPMI-1640 media plus 10% fetal calf serum, with supplemental vitamins, nonessential amino acids, and HEPES buffer.

### Effector cells

VSV-N peptide–specific CTLs were maintained *in vitro* by weekly stimulation with the target VSV-N peptide, p52–59. Briefly, 4 × 10^5^ CTL clone 33 cells were incubated in a 24-well flat-bottom plate with 5 × 10^6^ irradiated (2,000 rads) B6 spleen cells plus 2 μM VSV-N p52–59 peptide suspended in RP-10 media. CTL clone 33 cells were analyzed for antigen-specific lytic reactivity with ^51^Cr-labeled N1 transfectant targets on day 5 and subsequently restimulated on day 7 of culture.

Alloreactive CTLs were induced by 1° stimulation of B6 spleen cells with irradiated (2,000 rads) spleen cells from BALB/c mice. Briefly, spleens were removed and processed into single-cell suspension preparations; BALB/c spleen cell suspensions were irradiated in a Gammacell 1000 cesium-137 irradiator (Atomic Energy of Canada Ltd., Kanata, Ontario, Canada) to deliver 2,000 rads. For 1° alloreactive stimulation, 25 × 10^6^ B6 spleen cells per flask were added to upright T-25 flasks with 25 × 10^6^ BALB/c irradiated spleen cells in 10 mL RP-10 media. Alloreactive cultures were placed in a 37°C humidified incubator at 7% CO_2_ for 7 days.

Secondary alloreactive cultures were prepared similarly in RP-10 media except that 2.5 × 10^6^ 1° effectors per flask were added together with 25 × 10^6^ irradiated BALB/c spleen cells to upright T-25 flasks. Cultures were incubated for 7 days in the same manner as the 1° alloreactive cultures. Subsequent cultures beyond the 2° alloreactive effectors were maintained in 24-well dishes (Corning-Costar; Corning Life Sciences, Corning NY,) by the addition of 1 × 10^5^ effectors plus 1 × 10^6^ irradiated BALB/c spleen cells per well in 2 mL RP-10 media supplemented with 5% rat concanavalin A supernatant as a source of interleukin-2.

### Mixed lymphocyte reaction assay

To measure the extent of alloreactive T-cell stimulation in mixed lymphocyte cultures (MLCs) and the effect of adding PRN on the induction of alloreactive CTL effectors, we used the mixed lymphocyte reaction (MLR) assay, as previously described (Sheil et al. 1987). T-cell proliferation was determined in a one-way MLR assay on day 4 of culture by the incorporation of tritiated thymidine (^3^H-TdR) by proliferating T cells. Briefly, after 72 hr of culture, 5 × 10^5^ viable 1° MLC cells in 100 μL plus 1 μCi ^3^H-TdR in 100 μL RP-10 were added per well to four wells per sample in a 96-well plate (Corning-Costar; Corning Life Sciences). After incubation for 18–24 hr at 37°C in a 7% CO_2_ humidified incubator, the cells were harvested, and the amount of proliferation was determined by measuring ^3^H-TdR uptake, as reflected by the total radioactive counts per sample in liquid scintillation fluid.

### ^51^Cr-release assay

We determined the lytic activity of peptide-specific and alloreactive effector CTLs using a standard 4-hr *in vitro* ^51^Cr-release assay, as previously described (Sheil et al. 1987). Briefly, tumor cells to be used as targets were labeled with radioactive sodium chromate (Na^51^Cr) and mixed with titrated doses of peptide-specific or alloreactive CTLs in 200 μL RP-10/well in 96-well round-bottom microtiter plates (Costar-Corning; Corning Life Sciences). The plates were incubated at 37°C in 7% CO_2_ for 4 hr and centrifuged, and 100 μL supernatant was collected from each well. The amount of specific lysis was determined according to the following formula: % specific lysis = (experimental release – spontaneous release) ÷ (maximum release – spontaneous release) × 100.

### PRN exposure

PRN (3,4-dichloroproprionaniline; > 97% purity) was purchased from Chem Service, Inc. (West Chester, PA) and dissolved in 70% ethanol (EtOH). Exposure of alloreactive effectors to PRN *in vitro* was accomplished by the addition of PRN concentrations of 16, 33, or 66 μM to the culture media at the initiation of culture (day 0) for 1° MLCs; for 2° MLCs, the PRN concentrations used were 66 and 165 μM. PRN remained in the media for the duration of the culture incubation period—usually 7 days. APCs and target cells were exposed *in vitro* to PRN (200 μM) for a period of either 18 hr or 2 hr at 37°C in 7% CO_2_, after which the PRN is washed out of the cultures.

### Statistical analysis

All results shown are representative of at least three repeated experiments, and the sample points in each experiment were run in triplicate. Thus, we performed all statistical analyses using triplicate samples for data points within each experiment. Statistical evaluation was conducted using the Student’s *t*-test analysis, and significance in observed differences as described in the text was established as being at a level of *p* ≤ 0.01.

## Results

To determine how PRN exposure might adversely affect cell-mediated immunity, we designed the initial experiments of this study to address its potential impact both indirectly (on *in vitro* antigen presentation to CTLs) and directly (on CTL lytic function). We first considered the requirement that antigen-specific CTLs must respond to a peptide antigen exposed on the surface of APCs bound to a self class I MHC molecule. If there is an adverse indirect effect on cell-mediated immunity due to PRN exposure, it could result from altered antigen processing and presentation characteristics of the APC. Alternatively, the exposure of potential effector CTLs to PRN might directly interfere with CTL proliferation and/or effector function. To address whether PRN exposure has a discernible effect on antigen presentation, we examined the well-characterized CTL response to the single antigenic peptide, VSV-N p52–59, in the context of the class I H-2K^b^ molecule (Sheil et al. 1987; Van Bleek and Nathenson 1990). To examine the possible direct effects on CTL proliferation and/or differentiation, B6 anti-BALB/c MLC-derived CTLs were used as alloreactive effectors.

### PRN-exposed APCs are recognized efficiently by VSV-N peptide–specific CTLs

**Figure 1:**
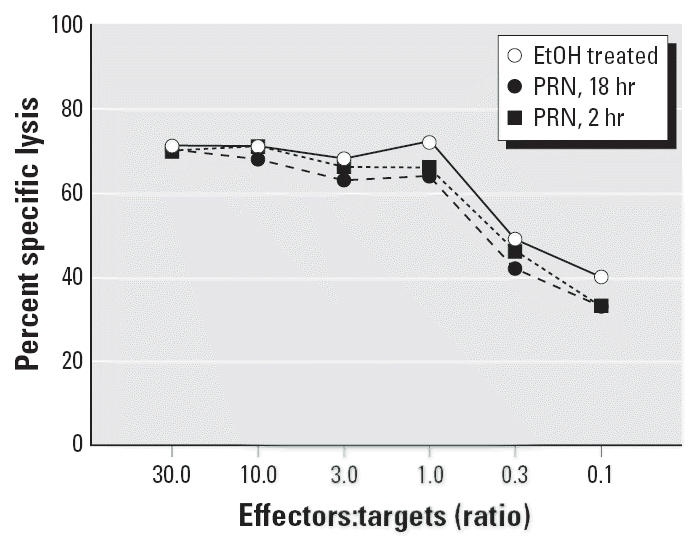
CTL clone 33 lytic response in a 4-hr ^51^Cr-release assay against N1 targets: EtOH treated, exposed to PRN for 18 hr, or exposed to PRN for 2 hr. The *x*-axis indicates the ratio of effector T cells per target cell added to each well in the ^51^Cr-release assay; ratios range from 30:1 to 0.1:1.

We examined the functional capacity of PRN-exposed APCs to determine whether PRN exposure of APCs *in vitro* adversely affects their ability to process and/or present antigen in the class I MHC pathway. The VSV-N transfectant model system (Puddington et al. 1986) was used, as described previously (Sheil et al. 1987), to determine whether exposure of VSV-infected cells to PRN interferes with their ability to effectively present viral peptide antigens to CTLs. In these experiments, CTL clone 33, specific for VSV-N p52–59 (Sheil et al. 1987), was tested against the target VSV-N transfected EL4 (H-2^b^) tumor cell line, designated N1. Initially, N1 cells were exposed to PRN for 18 hr before their use as CTL targets; however, because of undesirable levels of toxicity to the N1 cells (i.e., up to 30%), the period of incubation with PRN was decreased to 2 hr. After incubation in the presence of PRN for either 2 or 18 hr, N1 cells were tested as targets for lysis by clone 33. The results depicted in Figure 1 demonstrate that incubation of N1 cells with PRN does not adversely affect their capacity to serve as targets for lysis by VSV-N peptide–specific CTL effectors.

**Figure 2:**
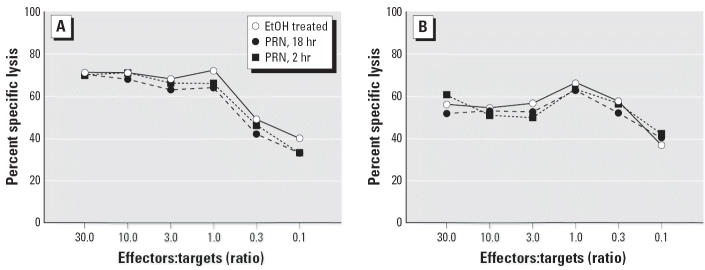
Clone 33 stimulated with EtOH-treated N1 (*A*) or PRN-treated N1 (*B*) stimulators, tested against N1 targets: EtOH treated, exposed to PRN for 18 hr, or exposed to PRN for 2 hr. Results shown are representative of three experiments; all sample points were run in triplicate. Effector:target ratios range from 30:1 to 0.1:1.

Another possible effect of PRN is its interference with the ability of N1 cells to serve as stimulators for the induction of VSV-N peptide–specific CTL effectors, even though they are undiminished in their capacity to serve as targets for CTL lysis. To address this possibility, we used N1 cells as APC stimulators for the VSV-N p52–59 peptide–specific, H-2K^b^-restricted CTL clone 33. After a 2-hr exposure to 200 μM PRN, N1 cells were added to culture flasks as stimulators for clone 33 CTLs. The 4-hr ^51^Cr-release assay results depicted in Figure 2 demonstrate that PRN-treated N1 cells are effective stimulators, in that they are undiminished, compared with control EtOH-treated N1 cells, in their capacity to stimulate lytic activity in clone 33 CTLs. This conclusion is reinforced by the observation that cell viability (as determined by trypan blue dye exclusion) and proliferative capacity (as determined by ^3^H-TdR uptake in the MLR assay) of clone 33 CTLs are not significantly different after culture with either control EtOH-treated or PRN-exposed N1 cells (data not shown).

### Citric acid–treated EL4 cells are recognized efficiently by VSV-N peptide–specific CTLs after PRN exposure

**Figure 3:**
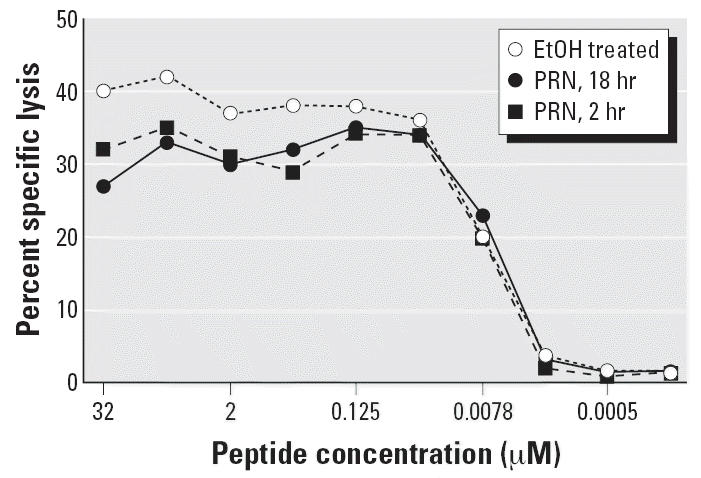
Peptide titration of clone 33 lytic response to VSV-N p52–59 tested on EL4 targets: EtOH treated, exposed to PRN for 18 hr, or exposed to PRN for 2 hr. A constant 3:1 effector:target ratio was used in this experiment. The *x*-axis indicates the peptide concentrations are titrated from 32 μM to 5 pM. Results shown are representative of three experiments; all sample points were run in triplicate.

Previous studies have demonstrated that a CTL effector requires only very few pMHC complexes on the surface of an APC to become activated by and lyse the APC as its target (Brower et al. 1994; Christinck et al. 1991). Thus, the ability of PRN-treated N1 cells to efficiently present antigen to CTLs and to serve as targets for lysis may correspond to their expression of this minimal number of pMHC complexes needed to target CTL lysis, even though it may be much lower than is normally expressed on N1 cells not exposed to PRN. To address this possibility, we subjected EL4 cells (from which the N1 cell line is derived) to citric acid treatment, an approach that strips most pMHC complexes from the cell surface (Storkus et al. 1993; Sugawara et al. 1987). As shown in Table 1, treatment of EL4 cells in this manner results in a similarly dramatic decrease in MHC class I expression, regardless of their subsequent exposure to PRN. This decreased MHC class I expression, however, does not adversely affect the presentation of peptide antigen to VSV-N peptide–specific CTLs, as shown by their undiminished lysis of acid-treated EL4 targets either with or without PRN exposure (Figure 3). Note that the exquisite sensitivity of this peptide-specific lysis is unaffected even with the addition of a concentration of target peptide as low as 78 pM. These results clearly indicate that the class I MHC antigen presentation pathway after PRN exposure, under these conditions, remains intact and fully functional.

### Primary alloreactive CTLs show limited inhibition after PRN exposure

Given that we observed no overt adverse effects of PRN exposure on APC function, we directed our attention to whether PRN exposure interferes directly with CTL function itself. To address this point, an alloreactive C57BL/6 (B6) anti-BALB/c mouse model system, as described in “Materials and Methods,” was used to obtain CTL effectors. The effect of PRN exposure on alloreactive CTL activation was examined in two ways. First, we measured the proliferative capacity of BALB/c-stimulated B6 CTLs in a standard ^3^H-TdR uptake assay. Second, to detect any functional changes in CTL effector activity, we measured allospecific CTL lysis of P815 targets in an *in vitro* 4-hr ^51^Cr-release assay (Sheil et al. 1987).

**Figure 4:**
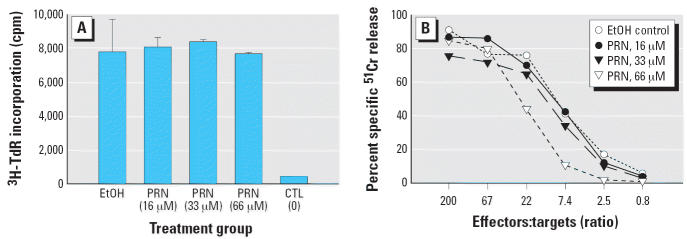
Primary B6 anti-BALB/c MLC-derived alloreactive effector CTLs tested against P815 (H-2^d^) target cells. MLC-derived effectors are from cultures with the following added on day 0: EtOH control, 16 μM PRN, 33 μM PRN, or 66 μM PRN. (*A*) Proliferation of MLC-derived T lymphocytes. The column designated “0” indicates that no CTLs were added to this group. (*B*) ^51^Cr-release assay results depicted as percent specific lysis by the following treatment groups: 0 PRN, 16 μM PRN, 33 μM PRN, or 66 μM). Results shown are representative of three experiments; all sample points were run in triplicate and represent mean ±SE.

The *in vitro* PRN-exposed 1° B6 anti-BALB/c CTLs are unchanged in their proliferative capacity compared with EtOH-treated control CTLs (Figure 4A), thereby indicating that there is no effect on their ability to proliferate in response to antigen stimulation after exposure to a range (16, 33, or 66 μM) of PRN concentrations. Furthermore, their functional lytic reactivity is only marginally inhibited after PRN exposure, and only at the highest (66 μM) concentration used in these experiments, as shown by the CTL lytic response in a 4 hr *in vitro* ^51^Cr-release assay (Figure 4B).

### Proliferation and reactivity of secondary CTLs are markedly impaired after PRN exposure

We next addressed whether the subsequent *in vitro* exposure of these alloreactive CTLs to PRN, during their 2° stimulation, might reveal an increased adverse effect on CTL proliferation and/or CTL lytic activity. To examine this possibility, 1° alloreactive CTLs were harvested on day 7 of culture, washed, and restimulated with the addition of fresh PRN as described in “Materials and Methods.”

**Figure 5:**
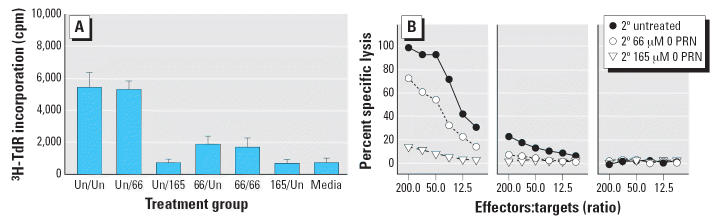
Secondary B6 anti-BALB/c MLC-derived effectors are tested for ^3^H-TdR uptake to measure the proliferative response (*A*) and ^51^Cr release as a measure of CTL-mediated lytic activity (*B*). Treatment groups in *A* are as follows: Un/Un, 1° EtOH-treated/2° EtOH-treated; Un/66, 1° EtOH-treated/2° 66 μM; Un/165, 1° EtOH-treated/2° 165 μM PRN; 66/Un, 1° 66 μM PRN/2° EtOH-treated; 66/66, 1° 66 μM PRN/2° 66 μM PRN; 165/Un, 1° 165 PRN/2° EtOH-treated. (*B*) ^51^Cr-release as a measure of CTL-mediated lytic activity. Treatment groups in *B* are as follows: 0 PRN; 2° 66 μM PRN; 2° 165 μM PRN. Results shown represent three experiments; all sample points were run in triplicate and represent mean ± SE.

In the same manner as with alloreactive CTLs from 1° MLCs, we tested 2° alloreactive CTLs for their proliferative capacity (Figure 5A) and their lytic responsiveness (Figure 5B). Secondary B6 anti-BALB/c MLCs were exposed to 66 or 165 μM PRN during *in vitro* 2° stimulation. Because we saw no effects at the lower PRN concentrations of 16 and 33 μM in 1° MLCs, they were excluded from further analysis with 2° CTLs. Instead, 2° MLCs were set up using the effective 66 μM PRN concentration, as well as a higher concentration of 165 μM.

We tested secondary CTLs on day 4 for proliferation (Figure 5A) and on day 5 for lytic activity against syngeneic P815 targets (Figure 5B). We included an important control group in which PRN-exposed 1° MLC effectors were washed and restimulated in 2° MLCs without additional exposure to PRN. As shown in Figure 5A, the proliferative capacity of the 1° EtOH-treated/2° 66 μM PRN group (second bar) is fully intact, whereas the 1° EtOH-treated/165-μM PRN group (third bar) shows no proliferative capacity above background (i.e., media control). Interestingly, for the lytic response of the 1° EtOH-treated/2° 66-μM PRN group (Figure 5B, left), the level of inhibition also is approximately double that seen with CTLs exposed to 66 μM PRN in 1° MLC (Figure 4B). On the basis of this observation, we are presently examining whether activated CTLs in 2° MLC might be more susceptible to PRN-mediated inhibition than are naive CD8^+^ T cells.

It is unlikely that the unresponsiveness observed in these groups is due to a generalized PRN-induced toxicity to the exposed CTLs because the viable cell yield of the 1° EtOH-treated/165-μM PRN group is approximately 70% of the EtOH-treated group, and that of the 1° EtOH-treated/2° 66-μM effectors is approximately 90% that of the EtOH-treated group. Furthermore, even the marginal decrease in cell viability observed in these groups has been taken into account in determining the total number of viable cells used in both the MLR and ^51^Cr-release assays. Cell populations used in both assays were equalized based on these total viable cell determinations; thus, the number of cells added is the same for each group. It is possible, however, that these cell viability determinations do not take into account damaged cells whose cell membranes are still intact because these cells would exclude the trypan blue dye until such point as membrane damage has occurred.

Another important, although initially unanticipated, finding concerns the control alloreactive CTLs in the 1° 66-μM PRN/2°-EtOH–treated group. These CTLs were initially exposed to 66 μM PRN during 1° stimulation, followed by restimulation in 2° MLC in the absence of PRN. The lytic activity of this group is almost completely ablated (Figure 5B, middle), even though the 1° response is only marginally inhibited compared with EtOH-treated 1° alloreactive CTLs (Figure 4B). Thus, the CTL lytic response of this group is nearly 20-fold lower in than that of 66 μM PRN-treated 1° CTLs.

To determine whether this unanticipated decline in CTL reactivity is irreversible, 3° MLCs were established without the addition of PRN as was done for the 2° MLCs. So, in this case we have alloreactive CTLs that have been activated multiple times, but they were exposed to PRN only during their initial activation. Among the 3° MLC-derived CTL effectors, both proliferation and lytic activity remained severely diminished (data not shown), as seen with the 2° CTLs. Thus, the profound PRN-induced defect incurred during their 1° MLC stimulation appears to render these CTLs irreversibly impaired.

## Discussion

Most herbicides and other pesticides exert a diverse array of immunotoxic effects on exposed individuals, including compromised humoral and cellular immunity (Banerjee et al. 1996; Rodgers 1995; Vial et al. 1996; Voccia et al. 1999). Earlier studies on the immunotoxic effects of PRN exposure by Barnett and co-workers (Barnett et al. 1992; Barnett and Gandy 1989) indicate that, although other important immune parameters are adversely diminished, the immunotoxic effects of PRN do not include impairment of cell-mediated immunity. This apparent anomaly in the immunotoxic impact of PRN exposure on different immune compartments prompted us to consider whether the effects of PRN on cell-mediated immunity might be more subtle or less easily detectable than effects on other immune compartments.

In the present study, we addressed the immunotoxic potential of the herbicide PRN on the effector cells of cell-mediated immunity, CTLs. A rigorous *in vitro* analysis of CTL activation and function was applied to determine whether and how PRN might induce immunotoxic effects in this regard. We approached this problem with the understanding that impaired cell-mediated immunity can result from the inhibition in antigen presentation to CD8^+^ T cells and/or, more directly, from a diminished functional CTL response.

Antigen processing and presentation defects have been implicated as the basis for impaired cell-mediated immunity induced by viruses (Fruh et al. 1997; Hewitt and Dugan 2004; Mylin et al. 1995) and by antioxidants (Gong and Chen 2003; Preynat-Seauve et al. 2003), as well as in tumor development (Bennink et al. 1993; Cohen et al. 2003; Restifo et al. 1993; Seliger et al. 1998) and aging (Plowden et al. 2004). The indirect consequences of these agents on antigen presentation can adversely affect the proliferation, differentiation, and effector functions of T lymphocytes—including cell signaling mechanisms, cytokine secretion, developmental maturation, and target cell lysis by CD8^+^ CTLs.

Thus it was important to examine the indirect immunotoxic effects of PRN exposure on the antigen processing and presentation component of cell-mediated immunity. In addition, the most common direct measures of CTL activation are proliferation and lytic activity. The experiments conducted in this study incorporate both indirect and direct approaches to determining the immunotoxic effects of PRN exposure on antigen presentation and CTL activation.

The most important findings of this study are that *a*) exposure to PRN during 1° CTL activation results in a dramatic delayed abrogation of CTL lysis that is irreversible, and *b*) the immunotoxic effects of PRN exposure under these conditions are limited to the functional activity of CTLs and do not affect antigen processing and presentation to CTLs. This study is unique in that it demonstrates such a striking difference between the short-term and delayed appearance of the immunotoxic effects of this herbicide. The issue of potentially delayed immunotoxic effects of pesticides has not been a focus of most studies, although some changes have been reported after *in utero* exposure that manifested after development (Colosio et al. 1999; Vial et al. 1996). This study, however, relates directly to the impaired activation of mature effectors of cell-mediated immunity. It is also important that these effects impair the proliferation and lytic activity of CTLs without interfering with the presentation of antigen by APCs.

In the initial approach to address whether PRN exposure inhibits cell-mediated immunity, we examined its possible impact on antigen processing and presentation in the class I MHC antigen presentation pathway, and indirectly on CTL induction and responsiveness. This approach used VSV-N gene-transfected N1 cells treated with PRN as targets for CTL-mediated lysis by CTL clone 33 (Sheil et al. 1987), which is H-2K^b^ restricted and specific for the VSV-N p52–59 peptide. Results depicted in Figures 1 and 2 reveal that exposure of APCs to PRN does not interfere with their ability to target CTL-mediated lysis in an antigen-specific manner. Nevertheless, it is possible that PRN could adversely affect the ability of APCs to effectively stimulate CTLs in culture. The results depicted in Figure 2 demonstrate that the responses between clone 33 CTLs stimulated with EtOH-treated (Figure 2A) or PRN-exposed (Figure 2B) N1 cells are similar in their responsiveness to N1 targets, indicating that PRN exposure also does not interfere with antigen presentation in a stimulatory capacity. Thus, the antigen presentation characteristics of N1 cells both as stimulators and as targets for clone 33 CTLs are unaltered by PRN exposure.

The absence thus far of any adverse effects of PRN exposure on antigen presentation, however, could be misleading because of the large number of potential pMHC complexes on N1 cells that can be engaged by the clone 33 T-cell receptors (TCRs). Previous studies have shown that minimally approximately 50–200 pMHC complexes need to be engaged for a CTL effector to lyse its target (Christinck et al. 1991; Sykulev et al. 1994); it is likely that many more pMHC complexes are formed and available for engagement by the clone 33 TCR on the N1 cells. Thus, if PRN exposure only partially interferes with antigen processing or presentation, its adverse effect may be masked under these *in vitro* assay conditions. To circumvent this problem, we replaced N1 cells as targets in the CTL lysis assay with untransfected EL4 cells plus titrated amounts of the target peptide VSV-N p52–59 (Figure 3). With the addition of lower peptide concentrations during this titration assay, fewer pMHC complexes will be formed. If there is a defect in antigen presentation, it should become apparent at the lower peptide concentrations.

As shown in Figure 3, we observed significant lysis even when a peptide concentration as low as 7.8 pM is added to EL4 targets, yet there is no significant difference in the level of activity in PRN-exposed groups compared with the EtOH-treated control group. With the higher concentrations of added peptide (up to 32 μM), the apparent difference between EtOH-treated and PRN-exposed EL4 targets is not significant. And even so, the critical point to be made is that at lower concentrations with fewer surface pMHC complexes formed, the sensitivity of the assay is much greater, and at peptide concentrations < 125 pM, the experimental and control groups are virtually indistinguishable (Figure 3). Thus, there is no observable effect of PRN exposure on *in vitro* antigen presentation, and we concluded that APCs exposed to PRN are unimpaired in their ability to serve both as stimulators and as targets for peptide-specific CTLs.

In the next phase of our study, we addressed whether there is a direct effect of PRN exposure on CTL reactivity alone. One potential complication with the peptide-specific CTL model system is that when peptide is added to *in vitro* cultures, it can bind to MHC molecules expressed on the surface of CTLs themselves as well as to those on the APCs, thereby complicating the interpretation of experimental results. To circumvent this problem we used an alloreactive B6 anti-BALB/c MLC model system to examine the effect of PRN on CTL proliferation and function.

In this model the alloreactive B6 CTLs respond directly to the allogeneic class I MHC molecules expressed on the BALB/c stimulator cells without the need for added peptide. As shown in Figure 4B, alloreactive CTLs exposed to PRN during 1° MLC are largely unaffected in their lytic reactivity, with only a limited decrease in reactivity observed at the highest (66 μM) concentration tested. Although there is a 2.5-fold difference between the response of the 66 μM PRN-exposed group and control CTLs, given the similarities in magnitude of their overall response, this apparent difference is probably minimal. We also noted the concomitant observation that the proliferative responses, as measured by ^3^H-TdR uptake in the *in vitro* MLR assay, among all three PRN exposure groups are not significantly different from the EtOH control group (Figure 4A). These findings are similar to those reported initially by Barnett and co-workers (Barnett et al. 1992; Barnett and Gandy 1989) and seem to support their suggestion that PRN might have very little, if any, effect on cell-mediated immunity.

We next examined the impact of prolonged PRN exposure on these alloreactive CTL effectors by adding fresh PRN during their restimulation in 2° *in vitro* MLCs. The overall effect of this longer-term exposure to PRN is the appearance of a significantly increased adverse effect on both CTL proliferation and lytic activity. Although PRN exposure during 1° MLC activation has only a limited effect on these parameters, the subsequent exposure to PRN during 2° MLC activation has a much greater adverse effect on CTL effectors. Thus, the exposure of 2° MLC-derived CTLs to 66 μM PRN induced an almost 70-fold greater inhibition than the same PRN concentration used in 1° MLCs. This finding establishes the importance of long-term symptoms in the diagnosis and management of immunotoxic effects resulting from exposure to environmental contaminants, particularly with respect to repeated or chronic exposure over an extended time period.

Even more striking is the nearly complete abrogation of CTL function of 2° CTLs that were exposed to PRN in the 1° MLC but not to additional PRN upon 2° stimulation. The nearly 8-fold difference in proliferation plus greater than 20-fold difference in lytic activity upon restimulation of PRN-exposed 1° CTLs into 2° CTL effectors indicates that some early PRN-induced adverse event(s) must have occurred during 1° activation that has a greater long-term functional impact on cell-mediated immunity. Thus, the studies reported here highlight the dramatic differences between acute and chronic exposure effects as an important consideration when assessing the immunotoxic potential of environmental agents. This often-neglected yet clinically important parameter could have significant diagnostic and treatment ramifications for the detection and management of pathologic anomalies associated with exposure to environmental contaminants. It is also worth noting that the delayed appearance of immunotoxic effects after PRN exposure in the 1° MLC might provide insight into questions of environmentally relevant doses or concentrations used in studies of various toxicants. This finding suggests that the lack of observable toxic effects might be due to a delayed onset in the appearance of such effects rather than to the administration of an inadequate toxic dose.

## Figures and Tables

**Table 1:** H-2K^b^ expression on acid-treated EL4 cells: mean channel fluorescence (MCF).

	No antibody		Y-3 mAb (anti-K^b^)		Anti-K^b^ mAb mix	
Cell treatment	MCF	Ratio	MCF	Ratio	MCF	Ratio
EtOH-treated EL4	2	—	56	1.0	61	1.0
Acid-treated EL4	3	—	31	0.52	37	0.58
Acid-treated EL4 + 1 hr at 37°C	2	—	31	0.54	35	0.56
Acid-treated EL4 + PRN exposed	2	—	38	0.67	37	0.59
